# Alterations in neural activation in the ventral frontoparietal network during complex magnocellular stimuli in developmental dyslexia associated with READ1 deletion

**DOI:** 10.1186/s12993-024-00241-2

**Published:** 2024-06-26

**Authors:** Sara Mascheretti, Filippo Arrigoni, Alessio Toraldo, Alice Giubergia, Chiara Andreola, Martina Villa, Valentina Lampis, Roberto Giorda, Marco Villa, Denis Peruzzo

**Affiliations:** 1 Department of Brain and Behavioral Sciences, University of Pavia, Piazza Botta, 6, Pavia (PV), 27100 PV Italy; 2 Child Psychopathology Unit, Scientific Institute IRCCS Eugenio Medea, Bosisio Parini (LC), Italy; 3 Radiology and Neuroradiology Department, Children’s Hospital V. Buzzi, Milan, Italy; 4 Milan Centre for Neuroscience (NeuroMI), Milan, Italy; 5 Neuroimaging Unit, Scientific Institute IRCCS Eugenio Medea, Bosisio Parini (LC), Italy; 6 Université Paris Cité, CNRS, LaPsyDÉ, Paris, France; 7 Department of Psychological Sciences, University of Connecticut, Storrs, CT USA; 8 The Connecticut Institute for Brain and Cognitive Sciences, University of Connecticut, Storrs, CT USA; 9 Yale Child Study Center Language Sciences Consortium, New Haven, CT USA; 10 Molecular Biology Laboratory, Scientific Institute IRCCS Eugenio Medea, Bosisio Parini (LC), Italy

**Keywords:** Developmental dyslexia, fMRI, *DCDC2*, Magnocellular hypothesis, Dorsal stream vulnerability

## Abstract

**Background:**

An intronic deletion within intron 2 of the *DCDC2* gene encompassing the entire READ1 (hereafter, READ1d) has been associated in both children with developmental dyslexia (DD) and typical readers (TRs), with interindividual variation in reading performance and motion perception as well as with structural and functional brain alterations. Visual motion perception -- specifically processed by the magnocellular (M) stream -- has been reported to be a solid and reliable endophenotype of DD. Hence, we predicted that READ1d should affect neural activations in brain regions sensitive to M stream demands as reading proficiency changes.

**Methods:**

We investigated neural activations during two M-eliciting fMRI visual tasks (*full-field sinusoidal gratings* controlled for spatial and temporal frequencies and luminance contrast, and *sensitivity to motion coherence* at 6%, 15% and 40% dot coherence levels) in four subject groups: children with DD with/without READ1d, and TRs with/without READ1d.

**Results:**

At the Bonferroni-corrected level of significance, reading skills showed a significant effect in the right polar frontal cortex during the full-field sinusoidal gratings-M task. Regardless of the presence/absence of the READ1d, subjects with poor reading proficiency showed hyperactivation in this region of interest (ROI) compared to subjects with better reading scores. Moreover, a significant interaction was found between READ1d and reading performance in the left frontal opercular area 4 during the 15% coherent motion sensitivity task. Among subjects with poor reading performance, neural activation in this ROI during this specific task was higher for subjects without READ1d than for READ1d carriers. The difference vanished as reading skills increased.

**Conclusions:**

Our findings showed a READ1d-moderated genetic vulnerability to alterations in neural activation in the ventral attentive and salient networks during the processing of relevant stimuli in subjects with poor reading proficiency.

**Supplementary Information:**

The online version contains supplementary material available at 10.1186/s12993-024-00241-2.

## Background

Developmental dyslexia (DD) is a complex, heritable, neurodevelopmental disorder characterised by impaired reading acquisition in spite of adequate neurological and sensory function, educational opportunities and average intelligence [1]. DD represents one of the most common neurodevelopmental disorders, affecting about 7% of school-age children across languages, and is often associated with undesirable secondary outcomes [2], as well as with negative social impact and economic burden [3]. Following earlier descriptions of high familial aggregation of the disorder [4], substantial heritability typical of a complex trait has been reported [5], with estimates across DD and related quantitative traits ranging from 0.18 to 0.72 [6]. As expected for a complex heritable disorder with heterogeneous genotype-phenotype association patterns, several DD risk genes have been identified [2, 7, 8]. Although they have not been found to be associated with DD-related traits by recent genome-wide association studies [9–17] and in a large cross-linguistic sample [18], such genes play a role in neurodevelopmental processes such as neuronal migration, neurite outgrowth, cortical morphogenesis, and ciliary structure and function [19].

Among these genes, *DCDC2* has been identified as one of the main risk genes for DD. Animal studies have shown that *DCDC2* encodes for a protein with two DCX domains that are known to be essential for neurite outgrowth, neuronal migration and ciliary functions [19]. Its total and partial inactivation may result in structural and functional neuronal alterations, as well as in behavioral deficits in motion perception, auditory processing, working memory, visuo-spatial memory, visual discrimination and long-term memory [19]. Moreover, *Dcdc2* knock-out mice showed altered spike-time temporal precision and heightened excitability, which may in turn cause local interference with transmission of visual information and an effective loss of samples at specific positions [20, 21]. Human studies have demonstrated that variations spanning *DCDC2* are associated with performance on reading and reading-related skills in both clinical [20, 22–34] and general population samples [35–39].

Research has increasingly focused on a naturally occurring intronic deletion of 2,445 bp within intron 2 of the *DCDC2* gene, which encompasses the entire READ1 within its breakpoints (hereafter, READ1d). READ1 is a regulatory element, as it could act as a modifier of *DCDC2* gene expression and thereby influence neuronal migration [37]. With some lack of consistency [26, 40], independent studies showed a significant association between READ1d and DD, and DD-related phenotypes [17, 22, 25, 27, 29, 33, 41]. While negative findings have also been reported [40], two independent studies provided initial evidence about the association between READ1d and visual motion perception underlying the magnocellular (M) stream in both subjects with DD [42, 43] and typical readers (TRs) [43]. Visual motion perception was proved to be a reliable endophenotype (EP) of DD [44], significantly mediating the pathway from DD risk genes to reading [45]. Taken together, these findings supported the dominant, albeit controversial [46, 47] magnocellular theory of DD [48]. This theory hypothesizes that DD is due to a multimodal sensory impairment in the processing of transient and dynamic stimuli [46, 49–51] which arises from a deficit along the neural pathways involved in the fast transmission and processing of sensory information [47, 52, 53].

Extant imaging genetics research of READ1d suggests that this genetic mutation is associated with both structural and functional alterations in both subjects with DD and TRs [41, 54–56]. In adult TRs, READ1d was significantly associated with increased gray matter volumes in numerous reading/language-related regions, especially in the left hemisphere [54]. Moreover, READ1d was associated with reduced white matter integrity in the left arcuate fasciculus and the posterior corpus callosum, regardless of reading impairment status. For subjects with DD, white matter integrity was reduced bilaterally in the inferior longitudinal fasciculus and anterior corpus callosum in those with READ1d compared to those without it [55]. Recently, it has been shown that perceptual impairments exist in subjects with DD and READ1d [42, 43]. These perceptual impairments are accompanied by specific white matter anomalies in primary visual pathways that have been implicated in motion perception (i.e., optic radiations and ventral tracts which provide input to and output from V1) [56]. In addition to structural findings, an fMRI study found a nominally significant, positive correlation between READ1d and the activation in the left paracentral lobule during processing of printed non-words [41]. Interestingly, the functional alterations associated with READ1d in the temporo-parietal cortex is consistent with the localization of structural correlates of the READ1d [54]. Taken together, these results seem to link READ1d with temporo-parietal anomalies observed in DD.

Building upon the previous results demonstrating an association between READ1d, psychophysical functioning in response to M stimuli, and structural and functional brain alterations, we predicted that READ1d should affect neural activations in brain regions sensitive to M stream demands as reading proficiency changes. To address this prediction, we measured neural activation during two well-established fMRI visual tasks, i.e., full-field sinusoidal gratings in which we simultaneously manipulated spatial and temporal frequencies and luminance contrast close to threshold levels, and sensitivity to motion coherence for both threshold and suprathreshold levels [57] in subjects with DD with/without READ1d and in TRs with/without READ1d. To the best of our knowledge, this is the first study to apply a hypothesis-driven approach to an imaging-genetic study [7]. In particular, our study aimed to identify the links connecting a putative functional genetic variant (READ1d) spanning one of the most replicated DD-candidate genes (i.e., *DCDC2*), neural activations during tasks sensitive to M stream demands, and reading skills.

## Methods

The protocol was approved by the Scientific Review Board and the Ethical Committee of the Scientific Institute IRCCS Eugenio Medea.

### Sample

Due to the population imbalance of the frequency of subjects with/without READ1d, we contacted all parents of children with READ1d, and we randomly selected subjects without READ1d until we could form two samples about the same size. Logistical and time constraints were followed in doing so.

Subjects with a clinical diagnosis of DD [1] were recruited from a sample of a genetic study cohort, which had been genotyped for READ1d (*N* = 930) [29]. Inclusion criteria at the time of recruitment for the genetic study were: (1) either accuracy or speed z-score ≤ -2.00 on one or more of the following tests: text reading; reading of single unrelated words; reading of single pronounceable pseudowords [58–61]; (2) a mean score of 7 or higher (i.e., z ≥ -1.00) between the weighted scores of the vocabulary and the block design subtests of the WISC-III [62]; (3) absence of other neuropsychiatric, neurological or sensorial disorders. One hundred and fourteen subjects had READ1d (one homozygous and 113 heterozygous; hereafter, DD+), while 816 subjects did not (hereafter, DD-) (allelic READ1d frequency = 0.078) [29]. Subjects were asked to participate in the present study if they were 10–18 years old, had no contraindications to MRI, and had normal or corrected-to-normal vision. Thirty-nine subjects (18 DD + and 21 DD-) met these inclusion criteria and both parents signed a written informed consent form to participate in the present study.

TRs were recruited *via* two different schemes. (A) children 10–18 years old with no contraindications to MRI, absence of other neuropsychiatric, neurological, or sensorial disorders, and normal or corrected-to-normal vision, were contacted by word of mouth among students attending middle and high schools. Parental written informed consent for the collection of the mouthwash-sample to obtain DNA was acquired from 124 subjects; 15 subjects (all heterozygous) had READ1d (hereafter, TR+), and 109 subjects did not (hereafter, TR-) (allelic READ1d frequency = 0.069). All parents of the TR + group were contacted, and 14 of them provided written informed consent to participate in the present study. Parents of 17 TR- matched to the TR + group for demographic variables, provided written informed consent to participate in the present study. (B) children were selected from a community-based cohort of Italian children who had been recruited for a larger study [63, 64] investigating the effects of both genetic and environmental risk factors on behavioural, cognitive and linguistic measures. For the present study, we selected the sub-sample of subjects recruited in one (*n* = 235) of the five school districts involved in the original study. Genotyping for READ1d was available for 194 out of 235 subjects, and yielded 43 TR+ (one homozygous and 42 heterozygous) and 151 TR-. Allelic frequency of READ1d in this sub-sample did not significantly differ from those of the other four school districts (data available upon request). We were interested in 96 subjects who (1) were 10–18 years old, (2) had no contraindications to MRI and normal or corrected-to-normal vision, and (3) were free from neuropsychiatric, neurological, or sensorial disorders. Parents of nine subjects (five TR + and four TR-) provided written informed consent to participate in the present study.

Overall, the final sample of the present study was composed of 79 subjects, grouped in 18 DD+, 21 DD-, 19 TR+, and 21 TR-. Forty-four of these subjects (two DD+, 20 DD-, two TR+, and 20 TR-) had been included in a previous study aiming to identify the brain regions sensitive to M stream demands and relevant to classifying the subjects as either DD or TRs [57].

### Neuropsychological assessment [57]

At the time of recruitment for the present study, all subjects were administered the following tests: (1) An estimate of IQ was yielded with the Vocabulary and Block-Design subscales of the WISC-III [62]; (2) Reading abilities were assessed by means of the Text reading test [58, 59], the Single Unrelated Words reading test, and the Single Unrelated Pseudo-words reading test [60]. Speed (time in seconds) [65] and accuracy (number of errors) were recorded and converted in z-scores [60, 61]. Since bivariate correlations (*r*) across reading tasks were substantial (mean *r* = 0.723, Additional Table 1a), a single reading score was obtained by averaging the z-scores from the six reading parameters (hereafter, Mean Reading); (3) Verbal working memory (VWM) was assessed by the Single-Letter backward/forward span and the Single-Digit backward/forward span [66]. Scores were computed relying on the number of accurate letters/digits recalled in the correct order for each string, and then converted into z-scores [66]. Bivariate correlations (*r*) were moderate within VWM tasks (mean *r* = 0.457, Additional Table 1b); therefore, we created a composite score by averaging each test within VWM; (4) Phonological awareness was assessed by the Nonword Repetition subtest [67]. Scores were computed based on the number of correctly repeated nonwords, and z-scores were obtained [67]; and, (5) The Conners’ Parent Rating Scales-Revised: Long version [68–70] was used to assess attention deficit hyperactivity disorder (ADHD) traits. For the current purpose, two subscales were considered: DSM-IV-inattention (DSM-IV-I) and DSM-IV-hyperactivity/impulsivity (DSM-IV-HI). Hand preference was assessed by the Briggs and Nebes Inventory [71]. Socioeconomic status was defined on grounds of parental occupation which was scored according to the Hollingshead nine-points scale, whereby a score ranging from 10 to 90 was assigned to each parental job, and the higher of the two scores was used when both parents were employed [72].

Table 1 summarizes the descriptive statistics of demographic and neuropsychological variables of the total sample and the four groups (DD+, DD-, TR+, TR-).

**Table 1:** Descriptive statistics of demographic and neuropsychological variables in the four study groups

		Total sample (*n*=79)				DD+ (*n*=18)				DD- (*n*=21)				TR+ (*n*=19)				TR- (*n*=21)				χ2 *p*-value
**Sex (Male/Female)**		50/29				15/3				15/6				7/12				13/8				0.024
**Handedness**	**Right-handed**	58				14				15				13				16				0.919
	**Left-handed**	10				3				3				2				2				
	**Ambidextrous**	4				1				1				0				2				
		**Mean**	**SD**	**Skewness**	**Kurtosis**	**Mean**	**SD**	**Skewness**	**Kurtosis**	**Mean**	**SD**	**Skewness**	**Kurtosis**	**Mean**	**SD**	**Skewness**	**Kurtosis**	**Mean**	**SD**	**Skewness**	**Kurtosis**	**ANOVA** ***p-value***
**Age**		13.447	1.823	0.142	-0.700	14.384	1.872	0.041	-0.359	14.147	1.496	-0.478	-0.131	12.364	1.622	0.139	-0.848	12.925	1.634	0.788	-0.824	0.001
**IQ** ^**†**^		11.804	2.434	0.413	-0.092	11.167	2.044	-0.107	-1.326	10.595	1.693	-0.335	-0.400	11.763	1.813	0.983	0.496	13.595	2.910	-0.530	-0.128	<0.001
**IQ, Vocabulary**		11.203	3.065	0.198	-0.572	10.611	2.993	0.187	-0.514	9.762	2.879	0.291	-0.452	11.579	2.457	0.597	0.152	12.810	3.156	-0.049	-1.138	0.008
**IQ, Block design**		12.506	2.877	0.422	-0.226	12.278	2.866	0.023	-0.576	11.333	1.461	-0.008	0.304	11.947	2.172	0.548	-0.581	14.381	3.667	-0.626	-0.286	0.003
**TR, accuracy**		-1.397	2.703	-1.879	4.130	-3.195	3.139	-1.870	3.785	-3.414	2.379	-1.541	2.606	0.361	0.390	-0.655	1.196	0.569	0.377	0.008	0.513	<0.001
**TR, speed**		-1.128	1.660	-0.394	-0.941	-2.567	1.275	0.009	0.156	-2.552	0.959	-0.001	-1.102	0.327	0.603	0.179	0.671	0.145	0.680	0.075	-1.199	<0.001
**SWR, accuracy**		-1.520	2.606	-1.171	0.512	-3.412	2.583	0.070	-1.408	-3.444	2.495	-0.986	0.853	0.460	0.490	-0.654	-0.007	0.234	0.554	0.044	-1.066	<0.001
**SWR, speed**		-1.853	2.583	-1.251	1.142	-4.194	2.474	-0.545	-0.180	-3.480	2.281	-1.404	2.647	0.114	0.562	-0.317	-0.138	0.003	0.575	-0.050	-1.051	<0.001
**SPWR, accuracy**		-0.842	1.773	-1.478	3.268	-1.953	1.403	-0.230	-1.007	-2.249	1.932	-1.720	4.643	0.340	0.634	-0.182	-0.810	0.449	0.469	-0.260	0.317	<0.001
**SPWR, speed**		-1.521	2.386	-1.447	2.180	-3.597	2.193	-1.579	1.477	-3.084	2.194	-1.712	2.996	0.215	0.552	-0.060	-0.928	0.252	0.642	-0.204	-0.604	<0.001
**Mean Reading**		-1.382	2.009	-0.967	0.549	-3.160	1.458	-0.414	-0.281	-3.037	1.564	-2.170	5.535	0.303	0.305	-0.415	-0.495	0.275	0.329	0.330	-1.049	<0.001
**SLFS**		-0.616	0.992	0.041	-1.100	-0.958	0.988	0.218	-1.109	-1.237	0.616	-0.277	-0.984	-0.307	0.959	-0.456	-1.190	0.050	0.855	-0.520	-0.957	<0.001
**SLBS**		-0.315	0.900	0.217	0.172	-0.658	0.892	-0.459	0.200	-0.676	0.778	0.853	2.000	0.213	0.722	0.323	-0.318	-0.060	0.908	0.712	-0.405	0.004
**SDFS**		-1.059	0.768	0.582	-0.084	-1.417	0.540	1.006	1.492	-1.368	0.647	0.428	-0.375	-0.813	0.855	0.500	-0.068	-0.628	0.741	0.143	-0.072	0.001
**SDBS**		-0.265	0.884	2.315	7.510	-0.528	0.596	1.283	1.484	-0.547	0.485	0.544	0.991	-0.067	1.183	3.266	11.634	0.093	1.018	0.826	-0.488	0.052
**VWM**		-0.564	0.683	0.822	1.684	-0.890	0.580	0.274	0.799	-0.957	0.416	0.276	0.071	-0.243	0.735	1.912	4.555	-0.136	0.592	0.259	0.595	<0.001
**SNWR**		-0.286	2.494	-0.854	0.346	-0.801	2.620	-1.065	1.056	-1.887	2.595	-0.054	-0.068	0.965	1.614	-0.470	-0.431	0.921	1.703	-1.137	0.559	<0.001
**ADHD**	**DSM-IV-I** ^**‡**^	54.280	11.264	0.683	-0.523	60.778	11.904	-0.173	-1.199	59.786	11.139	0.372	-0.538	50.857	8.576	1.124	0.887	46.303	6.027	0.989	0.134	<0.001
	**DSM-IV-HI** ^**§**^	49.167	9.153	1.017	0.458	55.667	11.581	0.197	-1.082	49.115	7.984	1.030	1.369	46.482	8.371	0.888	-0.155	46.077	5.566	1.443	2.444	0.003
**SES** ^**¶**^		59.671	18.821	0.008	-0.852	60.556	22.089	0.032	-1.428	55.000	17.905	-0.173	-0.235	61.316	17.388	-0.247	-0.470	61.429	18.516	0.240	-0.976	0.696

TR=Text reading; SWR=Single unrelated words reading; SPWR=Single unrelated pseudo-words reading; Mean Reading=Average score among the z-scores from TR, SWR and SPWR (both accuracy and speed); SLFS=Single letters forward span; SLBS=Single letters backward span; SDFS=Single digits forward span; SDBS=Single digits backward span; VWM=Average score among the z-scores from SLFS, SLBS, SDFS and SDBS; SNWR=Single non-word repetition

^†^ Mean score of the weighted scores of the vocabulary and block design subtests of the WISC-III (Wechsler, 2006)

^‡^ The DSM-IV-Inattention (DSM-IV-I) subscale of the CPRS-R: L (Conners, 1989; Conners et al., 1998; Nobile et al., 2007)

^§^ The DSM-IV-hyperactivity/impulsivity (DSM-IV-HI) subscale of the CPRS-R: L (Conners, 1989; Conners et al., 1998; Nobile et al., 2007)

^¶^ As estimated by father’s/mother’s employment (Hollingshead, 1975)

### fMRI task design and data processing [57]

#### MRI acquisition protocol

MRI data were acquired on a 3T Philips Achieva d-Stream scanner (Best, The Netherlands) with a 32-channel head coil. Visual stimuli were developed with Presentation® software (Neurobehavioral System Inc., Berkeley, CA, USA) and delivered through a VisuaStim digital device for fMRI (Resonance Technology Inc., Northridge, CA, USA). MRI-compatible goggles with two displays were used, with a 60 Hz frame rate and 800 × 600 spatial resolution (4/3 aspect ratio) subtending a horizontal visual angle of 30 degrees. An MRI-compatible pad was used to record subjects’ answers and response times. The MRI protocol included the use of an anatomical T1-weighted (T1W) 3D Turbo Field Echo sequence as a subject morphological reference of MRI data (Field Of View (FOV) = 256 × 256 × 175 mm^3^, voxel size 1 × 1 × 1 mm^3^, time of repetition (TR) = shortest (~ 8.1 ms), Time of Echo (TE) = shortest (~ 3.7 ms), Flip Angle (FA) = 8 deg). The fMRI data were acquired with a T2*-weighted Gradient Echo planar sequence (FOV = 240 × 240 mm^2^, voxel size = 3 × 3 mm^2^, slice thickness = 3 mm, slice gap = 0.5 mm, slice number = 39, TR = 2 s, TE = 26 ms, FA = 90 deg). fMRI sequences included five dummy volumes acquired at the beginning of the sequence to reach steady states.

#### fMRI task design. Full-field sinusoidal gratings

The task consisted of 14s blocks of M, P and blank stimuli (fixation point only). The M stimulus was a monochrome, low spatial frequency (0.5 cycles per degree (cpd)), high temporal frequency (15 Hz), high luminance contrast, full-field sinusoidal grating with sinusoidal counterphase flicker. The P stimulus was a high colour contrast red–green, high spatial frequency (2 cpd), low temporal frequency (5 Hz), low luminance contrast full-field sinusoidal grating with sinusoidal counterphase flicker. The blank stimulus was a grey screen of mean luminance. Both gratings were presented at one of six orientations (0, 30, 60, 90, 120 and 150) and changed to the next orientation every 2.33s. The protocol included 28 blocks (8 M, 8 P and 12 blank) presented in pseudorandom order with the constraint that the same stimulus type could not appear in adjacent blocks to minimize adaptation to the stimuli. A white, 0.2-deg fixation point appeared at the centre of the screen throughout the blocks. Subjects were instructed to maintain fixation throughout the run, and they performed an irrelevant target detection task during the M and P stimulus blocks to encourage them to do so. The target appeared for 300ms with an appearance probability of 50%, in a random position and at random times during the second half of each stimulus block. At the end of each block, the screen turned grey and subjects were asked to press the corresponding button on the response pad to answer questions (i.e., ‘Did the target appear?’—right button for ‘Yes’ and left button for ‘No’).

#### CM detection

Sensitivity to motion coherence was assessed for radial motion (expanding or contracting). The stimuli included 100 small dots (each 20 arcmin across), half white and half black, presented for 250ms on a mean-luminance grey background. A portion of dots drifted coherently at a speed of 10 deg/s (limited lifetime of 8 frames, frame rate 60 Hz), while the remainder were displayed in random positions on each frame. Three proportions of coherently moving dots were used (CML: Coherent Motion Level): 6%, 15%, and 40%. At the beginning of each stimulation block, a white 0.2-deg fixation point appeared at the centre of the screen for 0.5s and was followed by the 0.25s CM stimulus. Subjects were instructed to maintain fixation throughout the run and were actively engaged in performing a motion detection task by pressing a button on the response pad (i.e., right button for ‘expanding’ and left button for ‘contracting’ pattern). After the stimulus, subjects had 4s to answer the question and were asked to respond even when they could not detect the motion direction. After responding, a 4.25s inter-stimulus interval was added to let the BOLD signal return to the initial steady state. The protocol included 48 stimuli (8 repetitions for each combination of coherence level and motion direction) administered in a pseudorandom order with the constraint that the same coherence level could not appear in more than two adjacent blocks regardless of motion direction.

#### Anatomical MRI data analysis

T1W images were corrected for bias field intensity artifacts using the N4 algorithm [73]. Subsequently, FreeSurfer tools (http://surfer.nmr.mgh.harvard.edu/, version 6.0) were used to further process the T1W images following the recon-all processing pipeline. After the surface-based registration of the individual images to the fsaverage template, we used the template annotation according to the HCP-MMP1 atlas (https://figshare.com/articles/dataset/HCP-MMP1_0_projected_on_fsaverage/3498446) to project the atlas Regions of Interests (ROIs) onto the individual images. The HCP-MMP1 atlas was used to parcellate the cortical grey matter of each hemisphere into 180 ROIs on the basis of multi-modal MRI data acquired on 210 healthy subjects [74].

#### fMRI data processing

The fMRI data were processed following the FreeSurfer Functional Analysis Stream (FSFAST, version 6.0). The preprocessing pipeline included motion correction, slice-timing correction, resampling on the ‘fsaverage’ template, smoothing, and intensity normalization. Template resampling was performed by exploiting the subject T1W images as an intermediate step, and smoothing was performed using a 3 mm FWHM filter. Outlier volume detection was performed using in-house developed software tools [57].

For each subject, contrast maps were defined within each task. Regarding the full-field sinusoidal gratings, two contrast maps were specified, i.e., M stimulus *versus* Baseline (M-vs.-B) and P stimulus vs. Baseline (P-vs.-B). Three contrast maps were outlined for the coherent motion; one for each level of motion coherence, i.e., Coherent Motion Level 6% vs. Baseline (CML6-vs.-B), CML15-vs.-B, CML40-vs.-B. Finally, average contrast values were computed in each ROI following the HCP-MMP1 parcellation of the cortex.

### Statistical analysis

ROI mean value for each contrast map was entered as a dependent variable in a univariate General Linear Model including READ1d (+ vs. -) and sex as factors, while age, Mean Reading, IQ-Block Design, and DSM-IV-I were included as covariates (Table 1). In addition, the interaction between READ1d and Mean Reading (hereafter, READ1d*Mean Reading) was added in order to investigate whether the effects of READ1d upon neural activations during M-related tasks change as reading proficiency changes. We recruited both subjects with a clinical diagnosis of DD and TRs in order to cover the whole range of reading abilities; reading skill was analysed as a continuous regressor so as to maximise the extraction of information from the data. The critical threshold for identifying significant contributions of Mean Reading, READ1d, and READ1d*Mean Reading upon neural activations was set at *p* < 0.00014 (Bonferroni correction for 360 HCP-MMP1 atlas cortical regions).

## Results

### Planned analyses

**Fig. 1:**
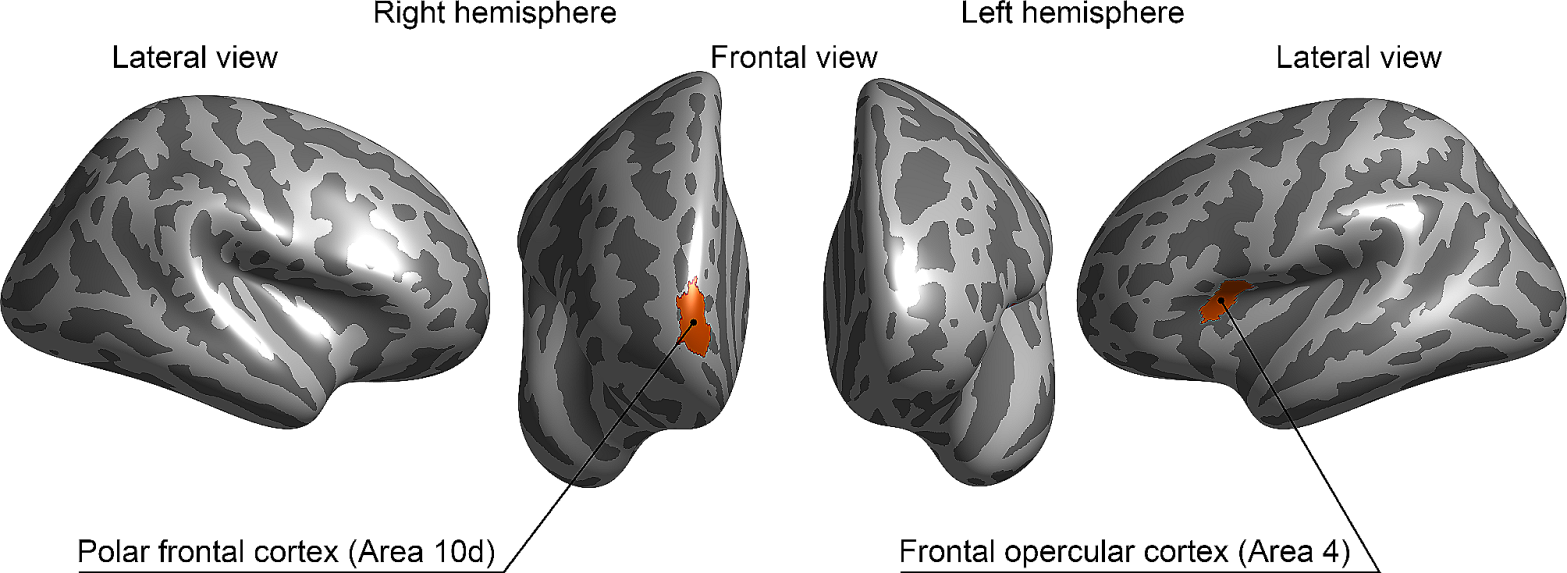
Contributions of Mean Reading, READ1d and READ1d*Mean Reading upon neural activations after Bonferroni correction for multiple comparison (*p* < 0.00014) The HCP-MMP1 atlas was used to parcellate the cortical grey matter of each hemisphere (Glasser et al., 2016). Significant Mean Reading (Right polar frontal cortex - Area 10d) and READ1d*Mean Reading (Left frontal opercular cortex - Area 4) effects were reported. No significant effects of READ1d were found

At the Bonferroni-corrected level of significance (*p* < 0.00014), two significant effects were found (Fig. 1).

**Fig. 2:**
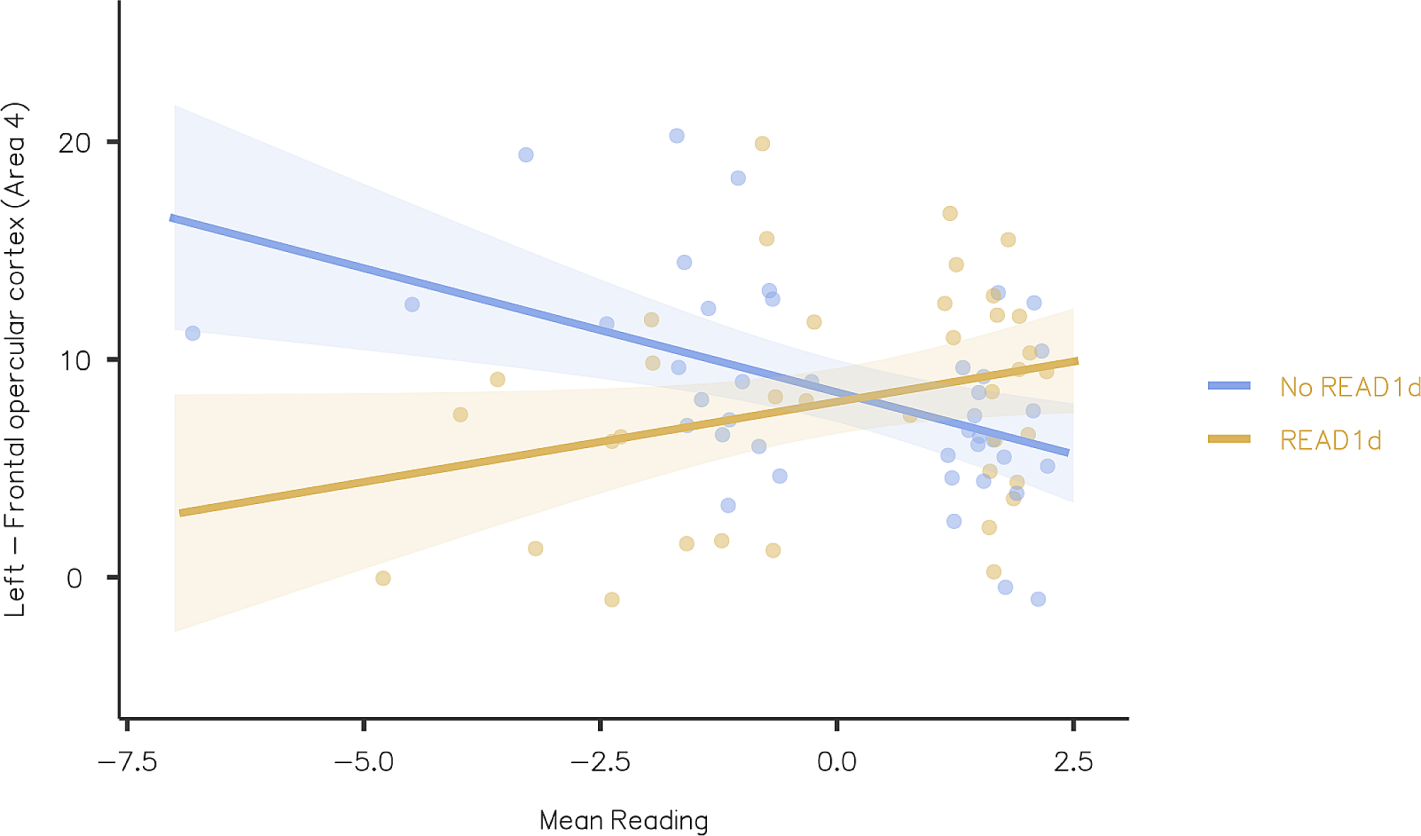
Contributions of READ1d*Mean Reading upon the left frontal opercular area 4 during the CML15 task Mean Reading = Average score among the z-scores from Text, Single Unrelated Words, and Single Unrelated Pseudo-words reading tests (both speed and accuracy); lower scores correspond to worse reading skills. 95% confidence intervals are reported. Significant READ1d*Mean Reading (Left frontal opercular cortex - Area 4) effects were plotted after Bonferroni correction for multiple comparison (*p* < 0.00014)

Firstly, reading skills showed a significant effect in the right polar frontal cortex during the M task (Table 2). Specifically, regardless of the presence/absence of the READ1d, subjects with poor reading proficiency showed hyperactivation in this ROI compared to subjects with better reading scores. Secondly, there was a significant READ1d*Mean Reading interaction in the left frontal opercular area 4 (insular and frontal opercular cortex) during the CML15 task (Table 2; Fig. 2).

**Table 2:** GLM analysis on the brain activation of the right orbital and polar frontal cortex during the M task

		M task				CML15 task			
		**Right - Polar frontal cortex (Area 10d)**				Left - Frontal opercular cortex (Area 4)			
		β	Standard error	p-value	η_p_²	β	Standard error	p-value	η_p_²
**Intercept**		1.262	0.289	< 0.001	0.214	5.579	6.354	0.383	0.011
**Sex**	**Male**	0.044	0.052	0.400	0.010	1.735	1.126	0.128	0.033
	**Female**	0.000				0			
**Age**		-0.005	0.001	< 0.001	0.203	-0.004	0.027	0.887	0.000
**IQ, Block design**		-0.027	0.009	0.005	0.109	0.334	0.201	0.101	0.038
**DSM-IV-I‡**		-0.010	0.003	< 0.001	0.169	-0.014	0.057	0.810	0.001
**READ1d**	**No READ1d**	0.065	0.063	0.146	0.030	-3.136	1.367	0.025°	0.070
	**READ1d**	0.000				0			
**Mean Reading#**		-0.087	0.021	0.00007	0.203	0.857	0.451	0.062	0.049
**READ1d**	No READ1d*Mean Reading	0.055	0.026	0.037	0.061	-2.294	0.562	0.00012	0.193
	READ1d*Mean Reading	0				0			

Regression estimates (β), their standard errors, *p-values*, and η_p_² are reported for all parameters of the model

° The direction of this effect is unexpected and is likely to be an artifact of the standard use of linear regression; however, it did not survive Bonferroni correction

^‡^ The DSM-IV-Inattention (DSM-IV-I) subscale of the CPRS-R: L (Conners, 1989; Conners et al., 1998; Nobile et al., 2007)

^#^ Mean Reading=Average score among the z-scores from TR, SWR and SPWR (both accuracy and speed)

Specifically, a reliable difference between the two genetic groups emerged among subjects with poor reading proficiency, suggesting that neural activation in this ROI during this specific task was higher for subjects without READ1d (Fig. 2, blue plot) than for READ1d carriers (Fig. 2, orange plot). Such a difference vanished as reading skills increased.

Sensitivity analyses were conducted using the G*Power software, Version 3.1.9.2 [75, 76], to compute the smallest effect size that our samples could detect with at least 80% statistical power. As a model, we selected “ANCOVA with fixed effects, main effects and interactions”; α was set at the Bonferroni-corrected level (0.00014), *N* = 79, power = 0.80. Under these assumptions, the minimal detectable effect size was 0.551.

### Additional analyses

**Fig. 3:**
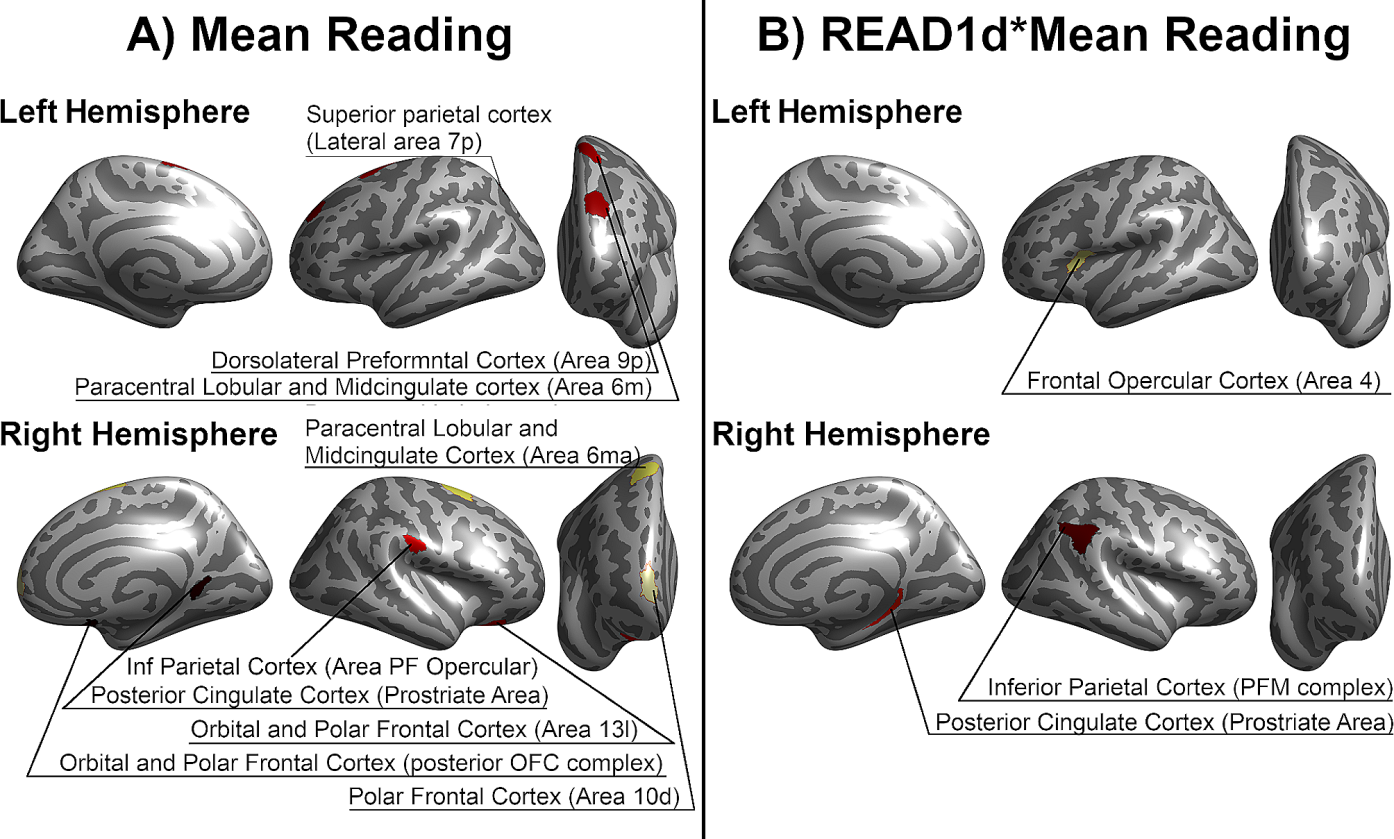
Contributions of Mean Reading, READ1d and READ1d*Mean Reading upon neural activations without Bonferroni correction (*p* < 0.001) The HCP-MMP1 atlas was used to parcellate the cortical grey matter of each hemisphere (Glasser et al., 2016). Significant Mean Reading (**Panel A**) and READ1d*Mean Reading (**Panel B**) effects were reported. No significant effects of READ1d were found

**Fig. 4:**
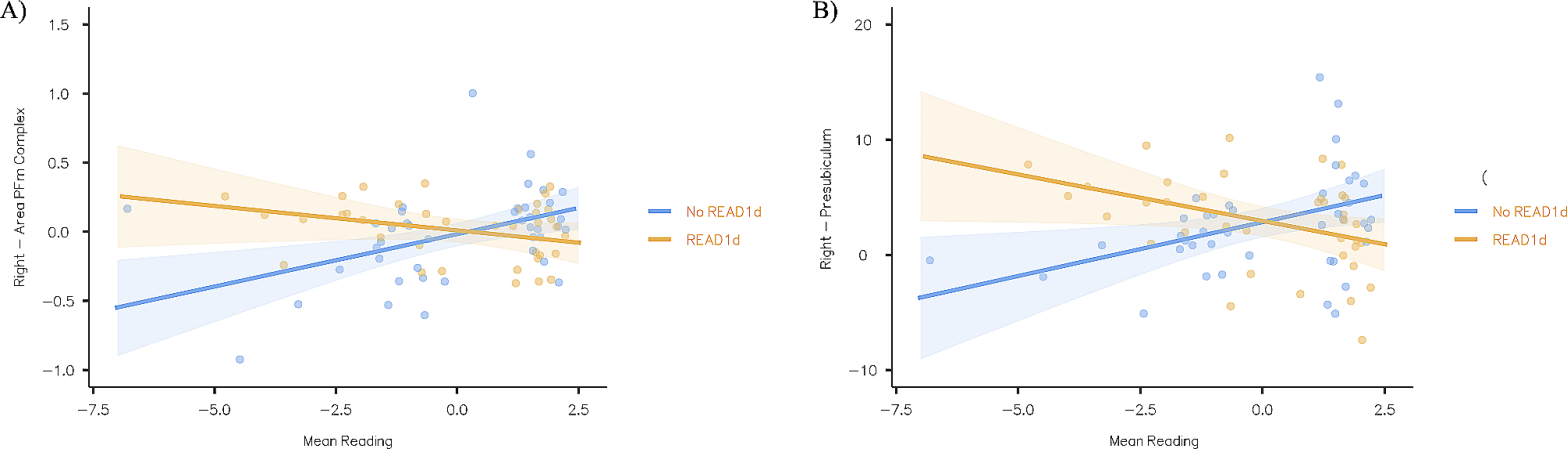
Contributions of READ1d*Mean Reading upon the right area PFm complex during the M task (**Panel A**) and upon the right PreSubiculum during the CML6 task (**Panel B**) Mean Reading = Average score among the z-scores from Text, Single Unrelated Words, and Single Unrelated Pseudo-words reading tests (both speed and accuracy); lower scores correspond to worse reading skills. 95% confidence intervals are reported. Nominally significant READ1d*Mean Reading effects were plotted (*p* < 0.001, uncorrected). Please, note that the significant READ1d*Mean Reading effect on left frontal opercular cortex - Area 4 was plotted in Fig. 2

For the sake of completeness, we also report results which turned out to be significant without Bonferroni correction (*p* < 0.001). Significant effects of Mean Reading, READ1d and READ1d*Mean Reading upon neural activation during the different tasks are reported in Additional Table 2, and 3. During the M and CML6 tasks, subjects with poor reading proficiency showed altered activation in the right posterior cingulate, frontal and inferior parietal cortices, in the left dorso-lateral prefrontal and superior parietal cortices, and in the bilateral middle cingulate cortex compared to subjects with better reading scores (Fig. 3 - Panel A). In addition, there were significant READ1d*Mean Reading interactions in the right area PFm complex (inferior parietal cortex) and in the right presubiculum (medial temporal cortex) during the CML6 task. A difference between the two genetic groups emerged among subjects with poor reading proficiency, but not in participants with better reading skills (Fig. 3 - Panel B and Fig. 4).

## Discussion

In this study we analysed in vivo evidence of differences in neural activation during M-stimuli processing linked to the READ1d-moderated genetic vulnerability and reading proficiency. Whereas genes are distal contributors, the brain is a more proximal driver of human behaviour; therefore, data on the putative functional pathways from candidate genes to behaviour are essential for the advancement of the field. Currently, there is a relative paucity of studies relating candidate genes, brain functioning, and behaviour in the developmental cognitive neurosciences, and in the DD literature specifically [41, 77–80]. To the best of our knowledge, this study provides the first piece of evidence for a hypothesis-driven approach [7] aimed at investigating the relationship between a putative functional genetic variant (READ1d) spanning one of the most replicated DD-candidate gene (i.e., *DCDC2*), neural activations during tasks sensitive to M stream demands, and reading skills.

Overall, our findings showed that, regardless of the presence/absence of READ1d, subjects with poor reading proficiency showed hyperactivation in the right polar frontal cortex compared to subjects with better reading scores during the M task. Moreover, our results showed that, among subjects with poor reading skills, READ1d significantly affected neural activations in the left frontal opercular cortex during the 15% CML, a stimulus known to require additional attentional resources for appropriate processing [42, 57, 81–83]. In particular, poor readers carrying READ1d had lower neural activation in the left frontal opercular area compared to their counterparts who lacked this functional genetic variant. Such a difference changes based on reading proficiency, with the gap closing as reading skills increase. Among subjects with high reading scores, no obvious differences in neural activations emerged between participants with and without READ1d. As to the apparent cross-over pattern visible in Fig. 2, we are unsure whether it reflects a real reversal of the effect for subjects with better reading scores (the effect did not survive Bonferroni correction; Table 2) or is an artefact of the use of a *linear* regression model. Indeed, if the real pattern involved curved plots converging at Mean Reading = 0 and remaining identical for Mean Reading positive values, the use of a (simple, standard) linear regression model would produce the cross-over pattern as a mere effect of under-fitting.

Taken together, these findings support previous work in which we reported that the M task elicits a set of areas lying within the dorsal portion of the M stream, the ventral attentive network (VAN) and the salience network, which discriminate between subjects with DD and TRs [57]. Although the pattern of activation did not completely overlap with the one previously described [57], possibly because of statistical and methodological issues (e.g., implementation of different statistical methods and analyses pipelines, or unbalanced prevalence of READ1d in the current samples), the current findings support the role of the dorsal portion of the M stream, of the VAN and of the salience network in DD. It is well-established that the ventral fronto-parietal network is linked to ‘dorsal stream vulnerability’ underlying several neurodevelopmental disorders, and that the functionality of the M pathway is intimately related to the systems involved in attention control [84, 85]. In other words, deficits in the M pathway could influence higher visual processing stages through the dorsal stream and, therefore, lead to reading difficulties through impaired attentional orienting [86–88]. The right frontoparietal system is a crucial component of the network subserving the automatic shifting of attention [89, 90], and developmental changes in its activation have been linked to reading skills in both children with DD [91, 92] and TRs [93]. Accordingly, alterations in M input processing in the dorsal visual stream and a consequent dysfunction of the main frontoparietal attentional network, are associated with a sluggish attentional shifting (SAS) [86] and a deficit in perceptual noise exclusion [82, 83] in DD [48, 52, 53, 94]. Irrelevant lateral letters should be filtered out by accurate and rapid shifts in spatial and temporal visual attention before the letter-to-sound mapping mechanism is applied [95–102]. This process may be more difficult if visual processing is hampered by deficits in attentional shifting and noise exclusion. Auditory and phonological impairments aside, spatial and temporal visual attention shifting and noise exclusion are crucially involved in forming representations that enable the efficient recognition of letters and letter sequences, the identification of word shapes and boundaries between words, the representation of sequential orthographic structure and the development of phonological representations [82, 83, 88].

In addition, our results are consistent with previous psychophysical findings suggesting that participants with good reading skills and READ1d may develop sensitivity to motion at both low and high spatial frequencies equal to that of good readers without READ1d [42]. According to the present findings and despite methodological differences, we can assume that the similar sensitivity to motion between good readers with and without READ1d could be explained by overlapping neural activations in brain regions spanning the ventral frontoparietal network while processing relevant stimuli [89]. Even though attention to stimulus attributes (e.g., sensitivity to motion) most consistently activates the dorsal frontoparietal network, neurophysiological and fMRI studies indicate that this network is also modulated by some properties of the salience maps in which top-down and bottom-up information interact to specify which relevant object to select [89]. By contrast, among participants with poor reading skills, we found a READ1d-moderated genetic vulnerability underlying neural activation within the frontal opercular cortex during a stimulus requiring additional attentional resources for appropriate processing (i.e., 15% CML). As for the M task, this level of coherently moving dots was previously found to be one of the most likely to elicit the most discriminating activations between subjects with DD and TRs in the dorsal portion of the M stream, the VAN and the salience network [57]. Moreover, we previously demonstrated that subjects (both children and young adults) with DD and READ1d were impaired in psychophysical tasks tapping the M stream compared to their counterparts without READ1d [42, 43]. The present findings suggest that differences in processing sensory relevant stimuli between poor readers with and without READ1d could be explained by a higher vulnerability among the former sub-group in the ventral frontoparietal network. This could in turn explain the large inter-subject variability in the M stream functioning observed in subjects with DD [46, 52, 86, 103–108].

The current study is not without limitations. First, although the EPs we used provide very sensitive measures of M function, thus increasing statistical power so results can be interpreted [109], the sample size of our study (*N* = 79) can still be considered relatively small, especially in light of the conservative multiple-comparison correction we used and of the results of the sensitivity analyses. Nevertheless, although it could have increased the probability of producing false negatives, the application of this very conservative multiple-comparison correction is justified to reduce the possibility of Type-I errors. In order to balance the risk of producing false negatives, to support our empirically-corrected findings and to formulate further hypothesis-driven investigations, we also reported results which turned out to be significant without Bonferroni correction (*p* < 0.001). Second, subjects with DD in our study might have had differences in brain activation as a consequence of lifelong poor reading, hindering our ability to make causal statements. Supporting this possibility is the recent meta-analytic data showing changes in brain activation from pre- to post- reading intervention in children with DD [110]. As a consequence, the use of subject-specific ROIs identified with *ad-hoc* experiments should be preferred over the population-based atlas approach we used in this study. Such an approach requires the implementation of function-specific fMRI tasks that should be administered to each subject during the MRI experiment [111, 112] leading to an increase of the acquisition time. This approach can therefore be implemented when focusing on few hypothesis-driven ROIs. As our study did not have a-priori hypotheses about specific ROIs, a subject-specific definition of functional ROIs was impossible. Third, limited by their correlational nature, neuroimaging genetic approaches cannot directly investigate causal mechanisms of DD in humans; however, these methods are key in building theoretical models of the etiologies of these prevalent neurodevelopmental disorders [7, 113, 114].

## Conclusions

Overall, we provided a piece of evidence about a READ1d-moderated genetic vulnerability to alterations in the neural activation of a cortical region implicated in the VAN and salience network during the processing of relevant stimuli in subjects with poor reading proficiency. Research investigating the relationship between candidate genes and clinical outcomes through the use of imaging derived functional EPs has the potential to optimise the criteria to diagnose DD and thereby facilitate the early identification of children with genetically-driven susceptibility. This could potentially develop into adequate and well-timed prevention strategies and the implementation of novel, evidence-based remediation approaches targeting impairments in specific reading-related EPs. These insights will aid in the earlier detection of children with DD and help improve their overall academic success.

### Electronic supplementary material

Below is the link to the electronic supplementary material.

Supplementary Material 1

## Data Availability

The datasets used and analysed during the current study are available from the corresponding author on reasonable request.
